# Intestinal Tuberculosis Presenting with Gastrointestinal Bleeding in Patient on Warfarin Therapy

**DOI:** 10.1155/2022/9277789

**Published:** 2022-05-14

**Authors:** Werimo Pascal Kuka, Joe Rakiro, Joseph Gatheru, Felix Riunga, Allan Rajula

**Affiliations:** Aga Khan University Hospital Nairobi, 4th Floor East Tower Block, Third Avenue Parklands, P.O. Box 30270, Nairobi 00100, GPO, Kenya

## Abstract

**Background:**

Intestinal tuberculosis (ITB) constitutes less than 5% of overall cases of extrapulmonary disease and mostly affects the ileocecal region. The presentation and radiologic findings in enteric tuberculosis can mimic Crohn's disease (CD). *Case Presentation*. We present a case report of an African woman who presented to a Kenyan hospital with lower gastrointestinal bleeding while on anticoagulation for valvular atrial fibrillation, and was diagnosed with intestinal tuberculosis after colonoscopy, biopsy, and positive staining for tuberculous bacilli.

**Conclusion:**

Intestinal tuberculosis causing gastrointestinal bleeding is rare but should be suspected in patients living in TB endemic regions.

## 1. Introduction

Tuberculosis (TB), an infection caused by *Mycobacterium tuberculosis*, is the tenth leading cause of death worldwide and the most common caused by a single infectious agent in the pre-COVID-19 pandemic era. The World Health Organization (WHO) Global Tuberculosis Report of 2020 estimated that there were 10 million cases of active tuberculosis worldwide and 1.2 million deaths in the preceding year. 90% of the infections were in thirty high TB burden countries including Kenya [1]. The country has an estimated TB prevalence of 558 per 100000 adults [2].

The primary infection site in most cases is the lungs, but virtually any other organ can be affected, which is referred to as extrapulmonary TB (EPTB). Abdominal involvement can be in solid organs, peritoneum, intestine, or lymph nodes. Intestinal tuberculosis (ITB) constitutes less than 5% of overall cases of EPTB cases, and although, it can involve the entire gastrointestinal tract (GIT), the most common sites of involvement are the ileocecal area, colon, and small bowel [3]. In contrast, the upper gastrointestinal tract, namely, the esophagus and stomach are rare sites of infection [4, 5]. Enteric TB can be a result of different modes of spread: direct infection by ingestion of bacilli from infected food or sputum, spread from an adjacent organ, and hematogenous or lymphatic spread [6].

The clinical presentation and diagnostic findings of ITB can mimic other infections, malignancies, and inflammatory bowel disease (IBD) [7]. Although infectious causes predominate in the developing nations, the prevalence of IBD is rising in these countries [8]. Crohn's disease (CD), one of the forms of IBD, shares many clinicopathologic features with GIT TB, and each can lead to misdiagnosis of the other with the risk of deleterious outcomes [9, 10]. This is compounded by the low sensitivities of tests used to ascertain the diagnosis of GIT TB [10, 11].

EPTB has been shown to have worse outcomes in treatment, compared to lung disease [5]. A source of concern in abdominal TB is the risk of complications including fistula formation, perforation, and intestinal obstruction [12]. It is essential that the diagnosis is made and appropriate treatment is initiated. Here, we describe a case of a young female who presented with gastrointestinal bleeding while on warfarin therapy for atrial fibrillation and rheumatic heart disease, whose workup including imaging, endoscopy, and histology established a diagnosis of ITB in the setting of active pulmonary disease. CD was considered a differential diagnosis during the investigation of the patient.

## 2. Case

**Figure 1:**
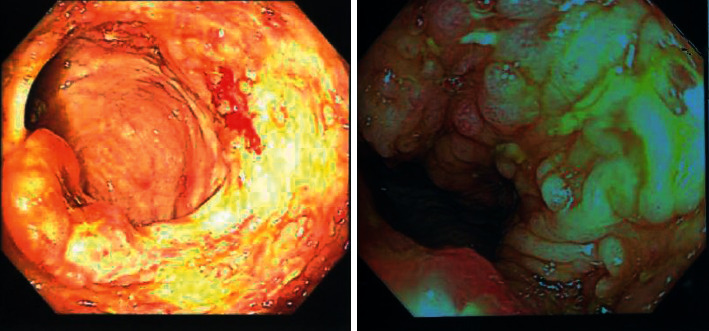
Colonoscopy: ulcerations and polyps in the ileocecal region and ascending colon.

**Figure 2:**
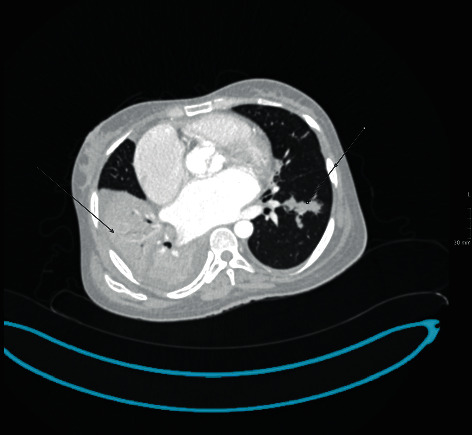
CT chest axial image showing bilateral lung consolidations.

**Figure 3:**
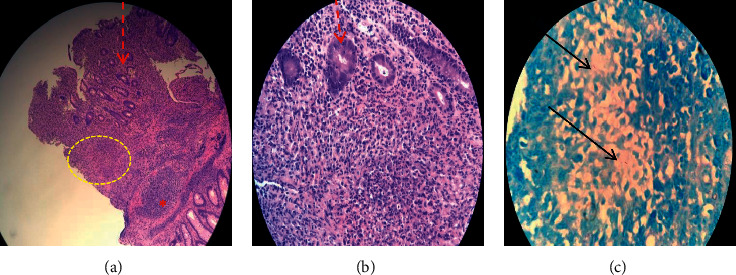
Colonic biopsies. (a) Lamina propria shows expansion by inflammatory cells. A lymphoid aggregate (red asterix), nonneoplastic colonic glands (red dashed arrow), and granuloma are seen (yellow dashed line) (hematoxylin and eosin, original magnification x100). (b) Goblet mucin component decreased as well as cryptitis. Edge of granuloma is seen here (original magnification x200). (c) Acid-fast bacilli (black arrows) (Ziehl–Neelsen stain).

A thirty-five-year-old female presented with complaints of per rectal bleeding mixed with stools. She had recurrent episodes associated with crampy lower abdominal pain over a period of five days. Upon further enquiry, she also reported a history of productive cough for three months, night sweats, and weight loss of ten kilograms over the same period. Her medical history was significant for rheumatic heart disease and atrial fibrillation. The patient had a surgical history of mitral valvuloplasty two years prior to her current presentation. Her regular medications included warfarin 5 mg daily, carvedilol 25 mg twice daily, and furosemide 40 mg daily. The examination revealed a sick-looking middle-aged female who had pallor, bipedal edema, and no palpable lymphadenopathy. The precordial examination was positive for features of mitral stenosis and aortic regurgitation. Abdominal examination did not elicit any tenderness or palpable mass. Investigations done included a complete blood count with the following results: white cell count 9.24 × 10^9^/L, hemoglobin 5.30 g/dL, and platelets 572 × 10^9^/L. The prothrombin time was prolonged, and the international normalized ratio (INR) was 2.78. Renal and hepatic function tests were normal. Initial management involved withholding of warfarin and transfusion of three units of packed red cells. An urgent colonoscopy performed to evaluate hematochezia revealed segmental inflammation of the ascending colon with ulcerations and polyps covered with yellowish exudate, a distended ileocecal junction, and an aberrant vascular pattern with mucosal edema extending to the hepatic flexure (Figure 1). At endoscopy, the differential diagnoses were tuberculosis or CD. In view of her respiratory complaints and accompanying systemic symptoms, a computed tomography (CT) of the chest and abdomen was performed, which demonstrated right lung consolidation, focal subsegmental consolidations in the left lung, and subcentimeter-sized lymph nodes in the small bowel mesentery (Figure 2). Given her history of chronic cough and chest imaging findings, a sputum test was requested whose staining was acid-fast bacilli (AFB) positive. Biopsies of the samples obtained during colonoscopy revealed lymphocytic infiltration, necrotizing granulomas, and positivity for AFB (Figure 3). Serologic testing for human immunodeficiency virus (HIV) was negative. A diagnosis of pulmonary and extra pulmonary tuberculosis with gastrointestinal involvement was established. The patient was initiated on the intensive phase of antituberculous therapy consisting of rifampicin (600 mg/day), isoniazid (300 mg/day), ethambutol (800 mg/day), and pyrazinamide (1 g/day). Warfarin 5 mg daily was reinstituted prior to her discharge after a five-day hospital admission. The culture in Löwenstein-Jensen medium later isolated *Mycobacterium tuberculosis* complex, which was susceptible to the first-line drugs. She is followed up at the outpatient clinic with regular INR checks to ensure a therapeutic INR range of 2.0-3.0. Her warfarin dosage was increased from the initial 35 mg/week to 52.5 mg/week by the fourth week after discharge.

## 3. Discussion

We present a case of ITB in a patient with concomitant pulmonary disease whose main presenting symptom was hematochezia while on warfarin therapy for atrial fibrillation and previous mitral valve surgery. The workup for an etiology of the gastrointestinal bleeding was pursued concurrently with that for her other complains identified on systemic enquiry. Although the patient is from a high TB burden country, a differential diagnosis of CD was considered during the initial investigations.

Extrapulmonary tuberculosis can be a manifestation of coexisting lung disease. The rate of coexistence of pulmonary and ITB has been reported to be in the range of 15–30% [4, 13]. The patient in our case had confirmation of active lung disease in addition to enteric TB. The likely mode of intestinal infection in such cases is ingestion of infected sputum.

The most common presentation of GIT TB is abdominal pain, which is reported by up to three quarters of patients [10, 13, 14]. Other symptoms include weight loss, fever, night sweats, and diarrhea. The patient in our case presented with hematochezia, which is a rare presentation of GIT TB, only seen in 7–20% of cases [10, 12, 13]. The cause of bleeding is postulated to be due to endarteritis caused by the bacilli [12]. A confounder in our case was the patient's use of warfarin. Mucosal inflammation due to infection is the likely major contributor to bleeding in our case. In a study to evaluate the etiology of major gastrointestinal bleeding in patients on anticoagulants, colitis was identified as the culprit lesion in 3% of patients on warfarin therapy [15]. Warfarin was resumed at discharge for our patient after a five-day admission duration. Reinitiation of anticoagulation after a clinically significant lower gastrointestinal bleeding is a challenging decision for clinicians because of the risk of recurrence of bleeding and the benefits of prevention of thromboembolism. Discontinuation of warfarin has been associated with an increased mortality in patients after admission for LGIB. The risk factors associated with mortality were age and medical comorbidities, in particular malignancies [16]. Our patient was young and had a low Charlson comorbidity index, therefore, placing her at low risk of adverse outcomes after the resumption of warfarin. The optimal timing for restarting warfarin is unknown and relies on clinical judgement of when the benefits outweigh the risks. In a review article, Kido and Scalese recommend resumption of warfarin therapy within 7–15 days after a major gastrointestinal bleed [17].

The constellations of symptoms of ITB are nonspecific, leading to either a long duration prior to diagnosis or misdiagnosis. In one report, the diagnosis of enteric TB was not considered initially in more than 60% of patients who had the eventual diagnosis [4]. The mimics include other infections, malignancies, and inflammatory bowel disease. CD was considered as a differential diagnosis during evaluation of our patient. Both ITB and CD have similar clinical, radiological, endoscopic, and pathological features, therefore, making it a challenging clinical problem [10]. In a report from China, in one of the high TB burden countries, up to 17% of patients with ITB were initially misdiagnosed to have CD [13]. Although TB is prevalent in the sub-Saharan African region, an increase in CD is also being reported [8]. It is crucial to identify the correct diagnosis because the use of immunosuppressive medications for CD can lead to flare up of TB with potential disastrous complications [18]. A meta-analysis showed the following features correlating with ITB more than CD: presentation with fever, night sweats, and lung disease. Other features include patulous ileocecal valve, cecal involvement, and presence of granulomas [19]. The patient in our case had all the above features favoring ITB over CD even prior to microbiological confirmation. In particular, the chest imaging findings which prompted sputum evaluation for TB was the key in our evaluation and should be performed in patients with colitis, especially in high TB endemic areas.

The colonoscopy finding of ileocecal involvement in our case was in keeping with other studies as the most common site in ITB [4, 6, 9, 20]. The reasons are postulated to be increased contact time between the mycobacteria and mucosa due to narrowing and stasis. Presence of lymphoid rich tissue in the ileocecal region, to which bacilli have a predilection for infecting, is also a possible contributory factor [21].

Biopsy of intestinal tissue for histology can be achieved by endoscopy as in our case. Other methods include percutaneously with imaging guidance, by endoscopic ultrasound, and surgically, either open or laparoscopic [6]. The demonstration of *Mycobacterium* in intestinal biopsy specimens can be by acid-fast staining, nucleic acid amplification, or culture. In addition, histologic findings of caseating granuloma are considered a hallmark in TB diagnosis [6]. The biopsy from patient in our case was positive for granulomas, AFB staining, and culture. PCR was not performed on the biopsy specimen. Although these tests are key in diagnosis, the sensitivity of any of the tests in ITB is low, with ranges of 5.3–37.5% for AFB tissue staining, 23–46% for mycobacterial culture, and 36.4–67.9% for PCR [19]. Because of the low sensitivities, a majority of patients in the high TB prevalence countries are only diagnosed retrospectively after successful empiric treatment with antituberculous medications despite initial extensive diagnostic investigations [9, 11, 13, 14].

The mainstay of treatment of ITB is medical therapy consisting of combination antituberculous therapy administered during intensive phase and maintenance phase. In drug susceptible TB, as in our case, the first-line medications during a two-month intensive phase are rifampicin, isoniazid, pyrazinamide, and ethambutol. The total duration of treatment is at least six months, though some advocate for extended durations of 9 or 12 months [4, 6]. The shorter course of treatment has been found to be just as effective as an extended period, with the added benefits of better compliance and savings on cost [22]. The role of adjuvant surgery is limited to complications such as intractable bleeding, obstruction, and fistulae [6].

A potential challenge in the outpatient follow-up of this case was the interaction between warfarin and rifampicin. Rifampicin is an inducer of cytochrome P450 hepatic enzymes which metabolize warfarin. Thus, there is a risk of inadequate anticoagulation in patients on both agents. It is recommended that INR be checked every two weeks in such cases and appropriate dose adjustments of warfarin be done to achieve a therapeutic INR.

## 4. Conclusion

ITB is a manifestation of abdominal spread of disease and should be suspected in patients presenting with gastrointestinal bleeding and constitutive symptoms in endemic areas. Although the clinical, radiologic, and endoscopic features are similar to CD, screening for pulmonary disease and microbiologic staining for specimens obtained during surgery, endoscopy, or radiologically can diagnose TB. The key treatment is a standard course of first-line antituberculous drugs.

## Data Availability

The dataset used to support the findings of this case report is included within the article and figures.
